# The New York Academy of Medicine

**Published:** 1890-12

**Authors:** 


The New York Academy of Medicine dedicated its new building, No. 17 West Forty-third street, on Thursday evening, November 20, 1890. The occasion was a memorable one in the history of the academy, and was celebrated with addresses, music and refreshments, that more than a thousand guests enjoyed until a late hour of the night.

Many physicians from a distance were present by invitation to take part in, or enjoy the ceremonies, among whom were Dr. S. Weir Mitchell, of Philadelphia; Drs. James R. Chadwick and R. H. Fitz, of Boston; Drs. A. Vander Veer, Franklin Townsend and M. J. Lewi, of Albany; Dr. B. F. Sherman, of Ogdensburg, N. Y.; Dr. J. O. Roe, of Rochester; Dr. J. S. Billings, of Washington, and Drs. H. R. Hopkins and W. W. Potter, of Buffalo.

The addresses were of an appropriate character, and the spacious hall was filled to its greatest capacity with the wealth, beauty, and fashion of the Metropolis, who listened attentively for two hours to the speakers. Afterward the floors were cleared, refreshments were served, and the remainder of evening spent in pleasant social intercourse.

The academy is to be congratulated upon its entrance into the new era in its existence that was so successfully inaugurated on this auspicious November night.

---

THE following additional appointments to the visiting staff of the Buffalo General Hospital have recently been made: Assistant Gynecologist, Dr. M. A. Crockett; Assistant Surgeon, Dr. John Parmenter; Assistant Physician, Dr. DeLancey Rochester.

